# My Teacher and Parents Make Me More Helpful Toward Others: The Sequential Mediating Roles of School Belonging and Prosocial Behavior in Adolescents’ Academic Achievement

**DOI:** 10.3390/children13081113

**Published:** 2026-08-20

**Authors:** Gözde Gümüşçağlayan, Gülşah Kıymık, Gökmen Arslan

**Affiliations:** 1 Department of Psychological Counselling and Guidance, Burdur Mehmet Akif Ersoy Üniversitesi, 15030 Burdur, Türkiye; 2 Department of Curriculum and Instruction, Burdur Mehmet Akif Ersoy Üniversitesi, 15030 Burdur, Türkiye; gulsahkiymik@mehmetakif.edu.tr

**Keywords:** parent–adolescent relationship, teacher–student relationship, school belonging, prosocial behavior, academic achievement

## Abstract

**Highlights:**

**What are the main findings?**

- Both parent–adolescent and teacher–student relationship significantly predicted adolescents’ school belonging, and prosocial behavior significantly predicted academic achievement. The relational predictors showed a differentiated pattern with respect to academic achievement: the parent–adolescent relationship had a significant direct association, whereas the teacher–student relationship showed no significant direct association.
- School belonging was related to academic achievement mainly indirectly—through prosocial behavior—rather than directly, indicating a primarily socio-emotional pathway. When tested directly, the full sequential pathway (relationship → school belonging → prosocial behavior → academic achievement) was statistically significant for teacher–student relationships but not for parent–adolescent relationships, indicating that this specific serial mechanism was supported only for the teacher–student pathway.

**What are the implication of the main findings?**

- Strengthening supportive relationships with parents and teachers may enhance students’ sense of school belonging and prosocial engagement.
- Because the full sequential pathway to academic achievement was supported only for the teacher–student relationship, and even then the effect was small and imprecisely estimated, supporting adolescents’ socio-emotional processes should be regarded as a plausible complementary direction rather than a firmly established route for improving academic outcomes; it is likely to be most meaningful alongside, rather than in place of, direct academic interventions.

**Abstract:**

**Background/Objectives**: This study used structural equation modeling (SEM) to examine the sequential mediating roles of school belonging and prosocial behavior in the effects of parent–adolescent and teacher–student relationships on adolescents’ academic achievement. **Methods**: Using a cross-sectional design, the study included 523 adolescents (61.6% female, 38.4% male) aged 13 to 19. Data were collected with the Mother–Adolescent Relationship Quality Scale, the Teacher–Student Relationship Quality Index, the School Belongingness Scale, and the Student Prosocial Behavior Scale, together with subjective (perceived achievement) and objective (school grades) indicators of academic achievement. **Results**: Both parent–adolescent (β = 0.25) and teacher–student (β = 0.44) relationships significantly predicted school belonging; school belonging significantly predicted prosocial behavior (β = 0.22); and prosocial behavior, in turn, significantly predicted academic achievement (β = 0.22). The direct effect of parent–adolescent relationships on academic achievement was significant (β = 0.26), whereas neither the direct effect of teacher–student relationships (β = 0.13) nor the direct effect of school belonging on academic achievement was significant. The indirect effects of both relational contexts on prosocial behavior via school belonging were significant, and school belonging predicted academic achievement indirectly through prosocial behavior; however, neither the total indirect effect of parent–adolescent relationships on academic achievement, nor its specific serial indirect effect through school belonging and prosocial behavior, reached statistical significance. The corresponding specific serial indirect effect for teacher–student relationships was statistically significant, although small in magnitude and not accompanied by a significant total indirect effect. **Conclusions**: These findings indicate that school belonging is more closely tied to socio-emotional processes than to academic achievement directly, and that the associations between relational contexts and academic achievement followed a differentiated rather than a uniform pattern: parent–adolescent relationships were linked to achievement primarily through a direct association, and their hypothesized distal indirect pathway to achievement via school belonging and prosocial behavior was not statistically supported. Teacher–student relationships, by contrast, showed no significant direct association with achievement, but the corresponding distal indirect pathway was statistically supported, although the effect was small and imprecisely estimated, underscoring the need for cautious interpretation of these relational mechanisms.

## 1. Introduction

Adolescence is a key period for identity development, social adjustment, and academic growth, and the parent–adolescent relationship plays a central role in this process. This relationship is built on parental warmth, emotional acceptance, open communication, and psychological support [1,2] and it shapes both the emotional and the academic development of adolescents [3]. Supportive, democratic parenting is consistently linked to academic achievement [4,5,6] and parental involvement is a well-established predictor of student achievement [7,8,9,10]. However, findings across this literature are not entirely uniform, and some studies report weaker or inconsistent associations between parental involvement and achievement depending on the outcome measure and cultural context [7]. Parental support also benefits social–emotional development: warmth and responsiveness strengthen prosocial tendencies [11,12] and help adolescents develop social skills such as empathy and cooperation [13,14,15]. Early experiences of attachment and trust within the family also provide a basis for school belonging [16,17,18], which in turn supports academic motivation and achievement [19,20,21]. For these reasons, the effect of parent–adolescent relationships on academic achievement is thought to operate mainly indirectly, through socio-emotional processes such as school belonging and prosocial behavior [22,23]. Consistent with this indirect view, strength-based parenting has been shown to relate to adolescents’ academic motivation through school belonging [24].

Although school belonging has frequently been linked to students’ academic motivation and functioning in prior research [19,21], supportive relationships with parents and teachers have also been associated with students’ prosocial behavior and academic motivation [25]. Building on these findings, the present study examines prosocial behavior as one potential socio-emotional mechanism through which relational contexts may be associated with academic achievement via school belonging. Given the cross-sectional nature of the present study, however, the proposed pathway should not be interpreted as strictly unidirectional or causal, and longitudinal research is needed to examine the directionality of these associations. Positive affect associated with academic success has been shown to broaden individuals’ thought–action repertoires, promoting greater cognitive flexibility, creativity, and openness to new ideas [26,27]. This suggests that academic success may contribute not only to cognitive development but also to the psychological resources that support constructive social interactions.

The teacher–student relationship is another key context that shapes adolescents’ school experience. When this relationship is marked by trust, emotional support, and low conflict [28,29], it strongly influences students’ attitudes and behavior toward school. Relationship quality is linked to long-term academic outcomes, and high conflict in particular is associated with academic and behavioral problems [30]. Teacher support increases academic interest, goal orientation, and motivation [25,31], and positive teacher relationships raise school satisfaction and engagement [31]. Research also confirms the role of the teacher–student relationship in student engagement and achievement [29,32]. This relationship is furthermore one of the strongest predictors of school belonging [33,34] and supportive teacher relationships promote prosocial behavior [35,36]. What relationships with parents and teachers appear to share is that both foster school belonging, which is central to the student’s school experience. In this sense, school belonging can be seen as the point where family- and school-based relationships converge [21,37,38].

While prior research has examined how family relationships [22,23] and teacher relationships [35,36] separately relate to adolescents’ school belonging and prosocial behavior, the present study extends this literature by testing a single sequential model in which school belonging and prosocial behavior jointly mediate the effects of both relational contexts. This integrative approach allows for a direct comparison of the relative contribution of family versus school relationships within one framework, which has not been consistently addressed in earlier work.

## 2. School Belonging

School belonging is the student’s sense of being accepted, valued, and supported at school [19,20], and it is central to how adolescents experience school [39]. It is more than emotional attachment; it also functions as a resource that supports academic engagement, self-regulation, and achievement [40,41]. Studies consistently show that school belonging is strongly linked to academic motivation, academic resilience, and achievement [17,19,21,42,43,44,45]. Relationships with parents and teachers are important for building this sense of belonging, because supportive relationships strengthen it [37,38,41]; indeed, parental emotional support and teacher factors emerge as significant predictors of school belonging within a socio-ecological framework [46]. A strong sense of belonging also encourages prosocial behavior and a more positive orientation toward others [23,47]. School belonging is therefore treated here as a key mediator linking relational contexts to academic and socio-emotional outcomes. School belonging is positioned prior to prosocial behavior in the proposed sequential model because feeling accepted, valued, and supported within the school community has been theoretically linked to students’ motivation, engagement, and broader behavioral functioning [19,20]. Accordingly, school belonging is conceptualized in the present model as a relational context that may facilitate subsequent prosocial engagement. However, this ordering should not be interpreted as establishing temporal precedence. Prosocial behavior may also contribute to the development of supportive social relationships and social acceptance [47]; therefore, alternative or reciprocal pathways between school belonging and prosocial behavior remain plausible and should be examined in future longitudinal research.

Attachment theory [48] provides a useful theoretical framework for explaining how relationships with parents and teachers foster school belonging. According to this theory, secure attachment experiences with caregivers in early childhood form internal working models in which the individual perceives the self as valuable and worthy of love [49], and these models eventually become a reference framework for interpreting social relationships [50]. The sense of belonging and acceptance that grows from secure attachment is thought to carry over to the school setting, helping school belonging develop [2,16]. In adolescence, this role is not limited to parents; teachers can also act as a secure base that gives adolescents a safe environment for exploration and growth [28,29]. From this view, positive relationships with both parents and teachers strengthen school belonging and the academic processes connected to it [21,25,40], so that relational experiences act as a bridge toward academic success. It should be noted that this theoretical account describes plausible directional processes rather than causal mechanisms established by the present cross-sectional data; the sequential model tested here therefore reflects a structural association consistent with attachment theory rather than a definitively causal pathway.

### 2.1. Prosocial Behavior

As attachment theory suggests, another outcome of secure relationships is a positive behavioral orientation toward others [48]. Prosocial behavior refers to voluntary positive acts such as helping, sharing, and cooperating, and it is closely tied to both social adjustment and academic processes [47,51]. Parental support and sensitivity strengthen prosocial tendencies [11,12] and teacher support is another important relational resource that promotes these behaviors [35,52]. A sense of school belonging likewise encourages prosocial behavior and increases students’ positive orientation toward their social environment [23,47]. From an academic perspective, prosocial behavior is positively associated with achievement at the meta-analytic level [47,53,54] and prosocial skills supported through social–emotional learning programs strengthen long-term academic adjustment [55]. Although previous research has demonstrated meaningful associations between prosocial behavior and academic achievement [47], the present sequential model should be understood as describing a theoretically plausible structural pathway among school belonging, prosocial behavior, and academic achievement rather than establishing a causal chain, given the cross-sectional nature of the present study. In this context, prosocial behavior is positioned as a key socio-emotional mechanism that grows out of school belonging and functions as a pathway toward academic achievement, which is itself a multidimensional outcome not fully captured by a single indicator.

### 2.2. Academic Achievement

Academic achievement is conceptualized as a multidimensional construct reflecting students’ performance within the school environment, and it is evaluated here in both subjective and objective terms. In its subjective dimension, academic achievement encompasses students’ perceptions and evaluations of their own performance; it develops in a reciprocal relationship with academic self-concept and mutually reinforces it over time [56,57,58,59]. This subjective assessment does not develop independently of the social context: teacher support, school climate, and peer relationships directly shape perceptions of academic competence [41,60,61]. In its objective dimension, the school report is regarded as a comprehensive indicator of success that reflects not only cognitive performance but also participation, responsibility, and behavioral adjustment [62,63]; self-control and academic perseverance strongly predict this indicator [62,64]. Across both dimensions, academic success appears to be shaped not only by individual characteristics but also by the social context: parental support [4,8], teacher relationship quality [29,65], school belonging [18,37] and prosocial behavior [47,54,55,66] are identified in the literature as key relational and socio-emotional factors in this process. Given these findings, understanding how these variables form a network of relationships—and how this network functions as a whole—becomes an important empirical question.

Although the literature documents robust bivariate links between parent–adolescent relationships, teacher–student relationships, school belonging, prosocial behavior, and academic achievement, studies that examine these variables holistically within a single model remain limited. The effects of parent–adolescent and teacher–student relationships on academic achievement have largely been examined in separate studies; models that test both relationship sources simultaneously are scarce. In addition, relatively little attention has been paid to sequential mediation models in which school belonging and prosocial behavior are positioned as sequential mediators, and studies that address both the subjective and objective dimensions of academic achievement together remain limited. This gap is not merely empirical but also conceptual: the sequential ordering proposed here—relational context, school belonging, prosocial behavior, academic achievement—follows directly from attachment theory [48], according to which secure relational experiences first foster a general sense of acceptance and worth (school belonging) before this sense of security manifests behaviorally in the individual’s orientation toward others (prosocial behavior); the present model therefore tests a theoretically derived sequence rather than one selected solely on the basis of prior empirical associations. The model proposed in this study addresses this gap by testing a sequential mediation structure in which the effects of parent–adolescent and teacher–student relationships on academic achievement are explained through school belonging and prosocial behavior. It is argued that supportive relationships enhance school belonging by reinforcing the adolescent’s perception of being accepted and valued at school [18,41], that school belonging encourages prosocial behavior [45], and that prosocial behavior supports both perceived and objective academic achievement [47]. Accordingly, this study aims to explain adolescents’ academic achievement not solely through individual characteristics but also through relational contexts—parent–adolescent and teacher–student relationships—and socio-emotional mechanisms—school belonging and prosocial behavior—by testing this network of relationships with structural equation modeling. As the model is tested with cross-sectional data, the hypothesized paths are best interpreted as theoretically grounded structural associations rather than as evidence of causal or temporally ordered effects.

### 2.3. The Present Study

The present study examines the sequential mediating roles of school belonging and prosocial behavior in the effects of parent–adolescent and teacher–student relationships on adolescents’ academic achievement, using structural equation modeling. The research is structured around a sequential mediation model predicting that parent–adolescent and teacher–student relationships influence school belonging, that school belonging influences prosocial behavior, and that prosocial behavior in turn influences both perceived academic achievement and report card grades. Within this framework, the following hypotheses were tested:

**H1.**  *Parent–adolescent and teacher–student relationships positively predict school belonging.*

**H2.**  *School belonging positively predicts prosocial behavior; parent–adolescent and teacher–student relationships also have direct effects on prosocial behavior.*

**H3.**  *Prosocial behavior positively predicts academic achievement; parent–adolescent and teacher–student relationships have direct effects on academic achievement.*

**H4.**  *School belonging mediates the effects of parent–adolescent and teacher–student relationships on prosocial behavior.*

**H5.**  *School belonging and prosocial behavior sequentially mediate the effects of parent–adolescent and teacher–student relationships on academic achievement (relationship → school belonging → prosocial behavior → academic achievement).*

These hypotheses reflect the directional relations implied by the theoretical framework outlined above; given the cross-sectional nature of the data, they were tested as structural associations within the proposed model rather than as claims of causal or temporally ordered effects.

## 3. Method

### 3.1. Participants

The study sample comprised 523 adolescents. Participants were selected from three secondary schools located in the central district of Burdur Province using a cluster sampling method. Schools were determined by taking into account administrative accessibility to the city center and representativeness in terms of student population size, and all classrooms within each school were included in the study. Data collection took place between April and May 2026. Of the 580 students invited to participate, 523 provided usable responses, corresponding to a response rate of 90.2%. Students were included in the study provided that they were enrolled full-time at the relevant school, voluntarily agreed to participate, and had parental/guardian consent; students who completed the questionnaire incompletely or who were enrolled in a special education program were excluded from the study. Given that participation depended on parental consent and students’ voluntary assent, some degree of self-selection bias may be present and is acknowledged as a limitation of the study.

An examination of the gender distribution revealed that 61.6% (*n* = 322) of the sample were female and 38.4% (*n* = 201) were male. Participants ranged in age from 13 to 19 years. The highest participation rate was observed in the 16-year-old group (27.9%; *n* = 146), followed by the 17-year-old group (25.8%; *n* = 135) and the 15-year-old group (25.0%; *n* = 131). Participants in the 14-year-old group accounted for 14.1% of the sample (*n* = 74), while the 18-year-old (6.1%; *n* = 32) and 19-year-old (0.8%; *n* = 4) groups were represented at lower rates; the smallest group was 13-year-olds (0.2%; *n* = 1). This distribution indicates that the sample consisted predominantly of adolescents in middle adolescence. Prior to data collection, approval was obtained from the Non-Interventional Clinical Research Ethics Committee of Burdur Mehmet Akif Ersoy University (GO 2026/2747, 2026-2), and the research was conducted in accordance with relevant ethical principles. The study commenced on 6 April 2026. Participation in the study was voluntary, and confidentiality and anonymity were ensured throughout the research process.

### 3.2. Measures

#### 3.2.1. Demographic Information Form

A demographic information form prepared by the researchers was used to collect background information and selected self-assessments. The first section collected information on participants’ age, gender, grade level, and grade point average for the most recent term. The second section asked participants to rate their satisfaction with school life (0 = very negative, 10 = very positive) and their level of loneliness (0 = not lonely at all, 10 = very lonely). Participants also rated their perceived academic achievement on a scale from 1 (very low) to 10 (very good).

#### 3.2.2. Parent–Adolescent Relationship Quality Scale

The Mother–Adolescent Relationship Quality Scale–Adolescent Form, developed by Wissink, Deković, and Meijer [67] and adapted into Turkish by Güre and Ergül Topçu [68], was used to assess parent–adolescent relationships. The scale comprises 18 items across three subscales: disclosure, positive parent–adolescent relationship (closeness and satisfaction), and negative parent–adolescent relationship (conflict and hostility). Items are rated on a 5-point scale (1 = not at all or very little, 5 = very much), with separate scores calculated for each subscale. Internal consistency coefficients reported for the Turkish adaptation were 0.84 for disclosure, 0.85 for positive mother–adolescent relationship, and 0.91 for negative mother–adolescent relationship [68]. Descriptive statistics and internal consistency coefficients for the present sample are reported in Table 1. Confirmatory factor analysis conducted with the current sample supported the three-factor structure, χ^2^(129) = 652.69, *p* < 0.001, χ^2^/*df* = 5.06, TLI = 0.907, CFI = 0.921, RMSEA (90% CI) = 0.088 (0.082, 0.095), with standardized factor loadings within an acceptable range. Although the RMSEA value falls slightly above, and the CFI value slightly below, the conventional cutoffs generally recommended for good fit [69], these indices are commonly regarded as reflecting acceptable, if marginal, fit for multidimensional scales administered in large field samples, particularly given the scale’s relatively large item count and the sensitivity of χ^2^-based indices to sample size; this marginal fit is nonetheless acknowledged as a limitation that may have introduced some measurement imprecision into the structural estimates involving this construct.

#### 3.2.3. Teacher–Student Relationship Scale

The Quality of Teacher–Student Relationships Index, developed within the Programme for International Student Assessment (PISA) framework, was used to assess the quality of teacher–student relationships. The eight-item scale measures students’ perceptions of teacher qualities such as respect, interest, warmth, and friendliness on a 4-point scale (1 = strongly disagree, 4 = strongly agree). The index is standardized such that the mean across OECD countries is zero and the standard deviation is one, with positive values indicating relationship quality above the OECD average.

Item-level inspection of the full eight-item scale revealed that two items—Item 4 (“I am wary of the teachers at my school”) and Item 8 (“The teachers at my school are rude to me”), both of which were negatively worded relative to the predominantly positively worded remaining items—showed negative corrected item-total correlations (−0.090 and −0.315, respectively), and that the internal consistency of the eight-item scale (α = 0.64) fell below the conventional 0.70 threshold. Removing either item would have raised alpha above this threshold (α = 0.713 and α = 0.756 if Item 4 or Item 8 were deleted, respectively), whereas the remaining six items displayed adequate corrected item-total correlations (0.472–0.601). Accordingly, Item 4 and Item 8 were excluded from all subsequent analyses, and the teacher–student relationship construct was assessed using the remaining six items throughout this study. Because this decision was based on item performance observed within the present sample, the resulting six-item measure reflects a data-dependent psychometric refinement rather than an independently validated scale revision; this limitation is discussed further in Section 5.2.

Descriptive statistics and the internal consistency coefficient for this six-item version in the present sample are reported in Table 1 (α = 0.84), indicating adequate reliability once the two psychometrically weak items were removed. Confirmatory factor analysis supported the unidimensional structure of the six-item scale, χ^2^(9) = 13.37, *p* = 0.147, χ^2^/*df* = 1.49, TLI = 0.993, CFI = 0.996, RMSEA (90% CI) = 0.030 (0.000, 0.062), with standardized factor loadings ranging from 0.63 to 0.74. These indices, however, describe the fit of a model estimated in the same sample from which the two weak items were selected for removal, and therefore do not constitute independent confirmation of the six-item structure. Taken together, these results indicate that the reduced reliability initially observed for the full eight-item scale was attributable to two specific items rather than to the construct as a whole, and the six-item version used in subsequent analyses should accordingly be regarded as a provisional measure pending replication in an independent sample (see Section 5.2), notwithstanding its adequate psychometric performance within the present dataset.

#### 3.2.4. School Belongingness Scale

The School Belongingness Scale [70] was used to assess sense of belonging at school. The ten-item scale comprises two subdimensions—acceptance at school (5 items) and exclusion at school (5 items)—rated on a 4-point scale (1 = never, 4 = always), with higher total scores indicating greater school belonging. Cronbach’s alpha for the full scale was 0.86, and the acceptance and exclusion subdimensions showed internal consistency values of 0.83 and 0.85, respectively. Descriptive statistics and internal consistency for the present sample are reported in Table 1. Confirmatory factor analysis supported the two-factor structure, χ^2^(34) = 150.95, *p* < 0.001, χ^2^/*df* = 4.44, TLI = 0.860, CFI = 0.894, RMSEA (90% CI) = 0.081 (0.068, 0.095), with factor loadings ranging from 0.40 to 0.68. Although internal consistency was satisfactory for both the full scale and its subdimensions, the fit indices for this measurement model were marginal by conventional standards, and the lowest standardized factor loading (0.40) indicated that at least one item captured a relatively limited proportion of the construct’s variance. This pattern of adequate reliability alongside marginal model fit suggests that, while the items reliably covary, the two-factor structure may not fully or optimally represent the underlying construct in the present sample; this measurement limitation is acknowledged and its potential influence on the structural estimates involving school belonging is discussed further below.

#### 3.2.5. Student Prosocial Behavior Scale

The Student Prosocial Behavior Scale, developed by Renshaw [71] and used with Turkish adolescents by Arslan and Tanhan [72], was used to assess prosocial behavior. The four-item scale measures positive social behaviors such as helping, respect, and cooperation displayed by the student in the school environment on a 4-point scale (1 = never, 4 = always); internal consistency was 0.79 in the validation study. Descriptive statistics and internal consistency for the present sample are reported in Table 1. Confirmatory factor analysis supported the unidimensional structure, χ^2^(1) = 3.07, *p* = 0.080, χ^2^/*df* = 3.07, TLI = 0.953, CFI = 0.992, RMSEA (90% CI) = 0.063 (0.000, 0.149), with factor loadings ranging from 0.39 to 0.67. As with the teacher–student relationship scale, internal consistency for this measure in the present sample (α = 0.64) was below the conventional 0.70 threshold and is addressed as a limitation below. Unlike the teacher–student relationship scale, item-level inspection did not identify a specific problematic item: all four items showed positive and reasonably comparable corrected item-total correlations (ranging from 0.396 to 0.452), and deleting any single item would not have raised alpha above its observed value. Composite reliability, calculated from the standardized factor loadings (0.39–0.67), was 0.62, which is comparable to the observed alpha rather than substantially higher; unlike the teacher–student relationship measure, this indicates that the reduced internal consistency of the present scale is not primarily an artifact of item count and more plausibly reflects genuine heterogeneity among items, with the item showing the lowest loading (λ = 0.39) contributing least to the shared construct. This measurement limitation is acknowledged, and its potential influence on the structural estimates involving prosocial behavior is discussed further below.

#### 3.2.6. Academic Achievement

Academic achievement was assessed across subjective and objective dimensions. Perceived academic achievement was a single-item self-assessment in which students rated their own academic performance on a scale from 1 to 10. Students’ average grades from their most recent term report card served as an objective indicator of academic achievement. Because these two indicators are expressed on different metrics and were not intended to be summed into a single composite score, descriptive statistics and correlations for perceived academic achievement and report card grades are reported separately in Table 1. In the structural model, academic achievement was specified as a latent variable indicated jointly by these two observed indicators—report card grade point average and perceived academic achievement—so that the construct would reflect both the objective and subjective dimensions of achievement simultaneously. Because a latent variable with only two indicators is not statistically identified when estimated in isolation, this measurement component was not tested as a stand-alone confirmatory factor model; rather, identification was achieved through its embeddedness within the larger structural model, where academic achievement was linked to the preceding relational and socio-emotional variables. This aspect of the measurement model is discussed further in relation to model fit in the Section 4.

### 3.3. Data Analytic Strategy

Data were analyzed using SPSS 27 and AMOS 22 Prior to the main analyses, the dataset was checked for missing values; no missing data were identified (N = 523 valid cases for all variables), so no missing data treatment procedure was required. Descriptive statistics (means and standard deviations) were computed for all study variables, and univariate normality was evaluated through skewness (g1) and kurtosis (g2) coefficients; these values generally fell within the ±2 range, indicating approximately normal distributions. Multicollinearity among the predictor and mediator variables was evaluated using variance inflation factor (VIF) values obtained from auxiliary regressions predicting each academic achievement indicator (perceived achievement and report card grades) separately; in both analyses, VIF values for all predictors ranged from 1.04 to 1.31, well below the conventional threshold of 5, indicating no problematic multicollinearity among the study variables. Multivariate normality was assessed using Mardia’s coefficient of multivariate kurtosis, which indicated substantial departure from multivariate normality (kurtosis = 120.921, critical ratio = 54.401). Point estimates for all structural parameters were obtained through maximum likelihood estimation. Given this departure from multivariate normality, bias-corrected bootstrap confidence intervals (5000 resamples) were computed for all indirect effects, including the specific three-path serial effects tested for Hypothesis 5, and are considered robust to this violation; direct path coefficients are reported with their associated maximum-likelihood-based critical ratios (t values), which should be interpreted with some caution given the observed nonnormality. Relationships among variables were examined using Pearson product–moment correlation coefficients. Given that data for all study variables were collected via self-report from a single source, common method bias was evaluated using Harman’s single-factor test, in which all 45 items from the study measures were entered into an unrotated exploratory factor analysis using principal axis factoring. The first, unrotated factor accounted for 21.23% of the total variance, well below the 50% threshold conventionally used to indicate the absence of a dominant common factor, suggesting that common method bias was unlikely to substantially threaten the validity of the findings [73].

A two-step approach was adopted to test the proposed model [74]. First, the construct validity of the measurement instruments was evaluated through confirmatory factor analysis (CFA); subsequently, structural relationships among the latent variables were examined using structural equation modeling (SEM). The two exogenous latent variables, parent–adolescent relationship and teacher–student relationship, were allowed to covary, consistent with the theoretical expectation that these relational contexts are naturally correlated. No post hoc modification indices were used to alter the hypothesized model, and no alternative structural models were tested; the model reported reflects the theoretically specified structure described above.

Model fit was evaluated using multiple indices: the ratio of chi-square to degrees of freedom (χ^2^/*df*), the comparative fit index (CFI), the Tucker–Lewis index (TLI), the incremental fit index (IFI), the goodness-of-fit index (GFI), the adjusted goodness-of-fit index (AGFI), and the root mean square error of approximation (RMSEA). Values of 0.90 or above for CFI, TLI, and IFI were interpreted as acceptable fit and values of 0.95 or above as good fit; for RMSEA, values of 0.08 or below were considered acceptable and values of 0.05 or below indicative of good fit [69,75].

The significance of the sequential mediation effects was tested using the bootstrap method, with bias-corrected 95% confidence intervals computed from 5000 resamples; indirect effects whose confidence intervals did not include zero were considered significant [76].

## 4. Results

Before testing the study hypotheses, descriptive statistics and Pearson correlations were examined for all study variables; results are presented in Table 1. Parent–adolescent relationships were positively and significantly correlated with teacher–student relationships (r = 0.16, *p* < 0.001), school belonging (r = 0.13, *p* = 0.003), and prosocial behavior (r = 0.14, *p* < 0.001). Teacher–student relationships were likewise positively correlated with school belonging (r = 0.38, *p* < 0.001) and prosocial behavior (r = 0.38, *p* < 0.001), and school belonging was positively correlated with prosocial behavior (r = 0.29, *p* < 0.001).

### 4.1. Descriptive Statistics and Correlations

Academic achievement was assessed through report card grades and self-reported perceived academic achievement. Report card grades of 85 and above were most prevalent in the sample. Perceived academic achievement, rated on a 1–10 scale, clustered predominantly at the moderate level, with ratings of 3 and 4 accounting for 79.1% of the distribution. The two indicators were only modestly correlated (r = 0.31, *p* < 0.001), suggesting that they capture related but partly distinct aspects of academic performance—one based on formal, teacher-assigned evaluation and the other on the adolescent’s own subjective appraisal.

### 4.2. Measurement Model

The fit of the measurement model was assessed via confirmatory factor analysis. The model demonstrated an acceptable overall fit, χ^2^(109) = 299.42, *p* < 0.001, χ^2^/*df* = 2.75. The comparative fit index (CFI = 0.927), Tucker–Lewis index (TLI = 0.909), and incremental fit index (IFI = 0.927) each met the proposed threshold values, and the goodness-of-fit index (GFI = 0.931) and adjusted goodness-of-fit index (AGFI = 0.903) similarly indicated adequate fit.

The root mean square error of approximation was 0.058, 90% CI [0.050, 0.066], which supports acceptable model fit; however, the associated PCLOSE value (0.048) falls just below the conventional 0.05 threshold, indicating that the hypothesis of close fit is only borderline tenable. Considered together, the fit indices indicate that the measurement model provides a generally acceptable, theoretically supported representation of the data, though this borderline PCLOSE value suggests some caution in interpreting the precision of model fit.

Academic achievement was specified as a latent variable with two observed indicators: perceived academic achievement and report card grades. For identification purposes, the loading of perceived academic achievement was fixed to 1.0, while the loading of report card grades was freely estimated (unstandardized λ = 10.758, SE = 2.343, C.R. = 4.592, *p* < 0.001). The standardized loadings were λ = 0.533 for perceived academic achievement and λ = 0.589 for report card grades, corresponding to squared multiple correlations (R^2^) of 0.284 and 0.347, respectively. The residual (error) variances were 0.644 (SE = 0.069, *p* < 0.001) for perceived academic achievement and 55.646 (SE = 7.349, *p* < 0.001) for report card grades—the latter reflecting the considerably wider metric of the 0–100 grading scale relative to the 1–10 perceived-achievement scale. Given the modest correlation between the two indicators (r = 0.31) and standardized loadings in the low-to-moderate range, these results should be interpreted with some caution: the two indicators appear to share a common academic-achievement factor while also carrying substantial indicator-specific variance associated with their distinct measurement approaches (objective, teacher-assigned grading versus subjective self-report).

### 4.3. Structural Model

**Figure 1:**
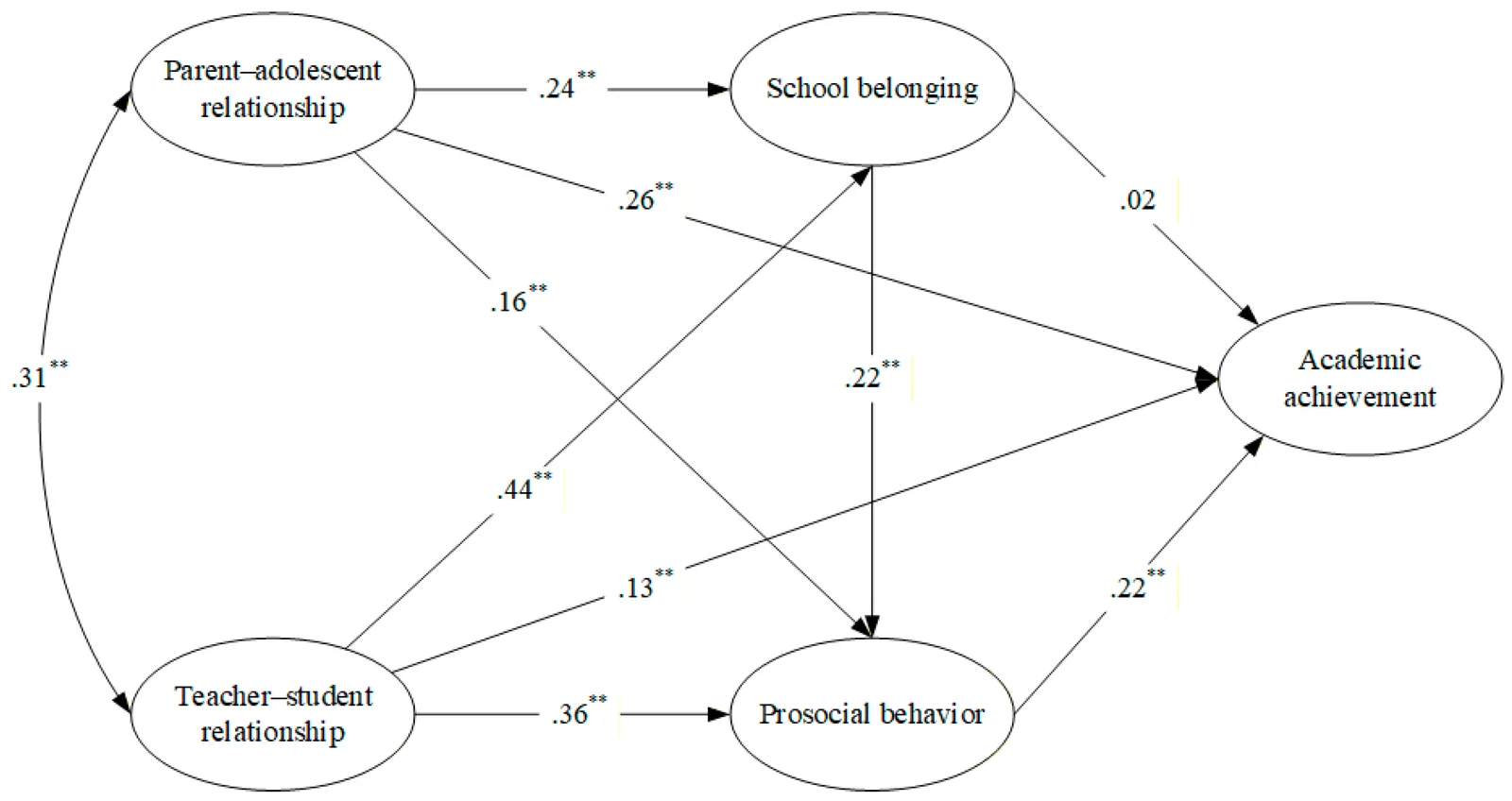
Proposed Structural Model. Note. ** *p* < 0.01.

The fit of the proposed structural model (Figure 1) was tested using structural equation modeling. The model demonstrated an acceptable level of fit, χ^2^(109) = 299.42, *p* < 0.001, χ^2^/*df* = 2.75, CFI = 0.927, TLI = 0.909, IFI = 0.927, GFI = 0.931, AGFI = 0.903, RMSEA = 0.058, 90% CI [0.050, 0.066], PCLOSE = 0.048. Because the structural component of the model is just-identified (saturated), its global fit indices are identical to those of the measurement model; the mediation hypotheses were therefore evaluated through the significance of individual path coefficients and bootstrapped indirect effects rather than through overall model fit. Direct path coefficients are reported below with their associated maximum-likelihood-based critical ratios (t values), whereas all indirect effects—including the specific serial effects central to Hypothesis 5—were evaluated using bias-corrected bootstrap confidence intervals, given the departure from multivariate normality noted in Section 3.3.

With respect to the direct effects within the structural model, parent–adolescent relationships positively and significantly predicted school belonging (β = 0.245, *t* = 4.218, *p* < 0.001), as did teacher–student relationships (β = 0.444, *t* = 7.203, *p* < 0.001). School belonging significantly predicted prosocial behavior (β = 0.223, *t* = 2.476, *p* = 0.013), and both parent–adolescent relationships (β = 0.158, *t* = 2.554, *p* = 0.011) and teacher–student relationships (β = 0.358, *t* = 4.696, *p* < 0.001) had significant direct effects on prosocial behavior.

With respect to academic achievement, prosocial behavior significantly and positively predicted academic achievement (β = 0.220, *t* = 2.046, *p* = 0.041), as did the direct effect of parent–adolescent relationships (β = 0.257, *t* = 3.136, *p* = 0.002). In contrast, the direct effect of school belonging on academic achievement was not significant (β = 0.019, *t* = 0.180, *p* = 0.857), nor was the direct effect of teacher–student relationships (β = 0.129, *t* = 1.377, *p* = 0.169).

Overall, these findings indicate a differentiated pattern rather than a uniformly indirect one. The parent–adolescent relationship was significantly and directly associated with academic achievement, whereas its total indirect effect through school belonging and prosocial behavior was nonsignificant (Table 2). Its specific three-path serial indirect effect, evaluated directly, was likewise nonsignificant (Table 3). The teacher–student relationship, by contrast, showed no significant direct association with academic achievement, and its total indirect effect through school belonging and prosocial behavior was similarly nonsignificant (Table 2); however, when the specific three-path serial pathway was evaluated directly, it reached statistical significance, although the effect was small in magnitude and imprecisely estimated (Table 3). These results therefore do not support the characterization that the relational variables affect academic achievement uniformly “primarily” through indirect pathways; rather, the two relational predictors appear to be linked to academic achievement through distinct mechanisms: the parent–adolescent relationship primarily through its direct association, and the teacher–student relationship, more tentatively, through the specific sequential pathway involving school belonging and prosocial behavior. The model accounted for 32% of the variance in school belonging, 35% of the variance in prosocial behavior, and 23% of the variance in academic achievement. The comparatively modest proportion of variance explained in academic achievement indicates that, beyond statistical significance, the practical magnitude of the relational and socio-emotional pathways examined here is limited; a substantial portion of the variability in adolescents’ academic achievement remains attributable to factors outside the present model, such as individual cognitive ability, prior achievement, or broader contextual influences.

Bootstrap analyses indicated that the mediation pattern proposed by the model was partially supported. The indirect effects of teacher–student and parent–adolescent relationships on prosocial behavior via school belonging were both significant (β = 0.099, 95% CI [0.025, 0.213], *p* = 0.011; β = 0.055, 95% CI [0.012, 0.136], *p* = 0.010). The indirect effect of school belonging on academic achievement through prosocial behavior was also significant (β = 0.049, 95% CI [0.001, 0.168], *p* = 0.044). Notably, the confidence interval for this latter effect approached zero at its lower bound, indicating a relatively weak and imprecisely estimated mediation effect that should be interpreted with caution rather than as strong evidence of a robust mediating mechanism. By contrast, the total indirect effects of teacher–student and parent–adolescent relationships on academic achievement were not significant (β = 0.109, 95% CI [−0.036, 0.269], *p* = 0.134; β = 0.051, 95% CI [−0.025, 0.137], *p* = 0.162). This pattern indicates that the proximal mediating chain formed by school belonging and prosocial behavior is operative, whereas the more distal total indirect pathway from relational contexts to academic achievement does not reach statistical significance; the specific three-path serial indirect effect central to Hypothesis 5 is evaluated separately below.

To evaluate Hypothesis 5 directly, the two specific three-path serial indirect effects were estimated as defined parameters and tested using bias-corrected bootstrap 95% confidence intervals based on 5000 resamples, applying the same criterion used for all other indirect effects in this study (i.e., a confidence interval excluding zero was treated as statistically significant). The serial pathway originating from teacher–student relationships was statistically significant, although small in magnitude (β = 0.027; unstandardized estimate = 0.024, SE = 0.017, 95% CI [0.004, 0.083]), whereas the pathway originating from parent–adolescent relationships was not (β = 0.016; unstandardized estimate = 0.002, SE = 0.001, 95% CI [0.000, 0.007]). Hypothesis 5 was therefore partially supported: the full sequential mechanism received statistical support for the teacher–student pathway but not for the parent–adolescent pathway. Two qualifications are warranted. First, the lower bound of the teacher–student interval lay close to zero and the point estimate was small relative to the corresponding total indirect effect, indicating a weak and imprecisely estimated effect rather than robust evidence of a substantial distal mechanism. Second, this specific effect was significant even though the total indirect effect of teacher–student relationships on academic achievement was not (Table 2), which is possible because the total indirect effect aggregates multiple routes of differing sign and precision; the two estimates are therefore not redundant, and the significant specific effect should not be read as evidence of a substantial overall indirect association between teacher–student relationships and academic achievement.

In relation to the study hypotheses, H1, H2, and H4 were fully supported, H3 was partially supported (prosocial behavior and parent–adolescent relationships, but not teacher–student relationships or school belonging, significantly predicted academic achievement directly). H5 was partially supported: the specific three-path serial indirect effect was statistically significant for the teacher–student pathway but not for the parent–adolescent pathway, even though the individual segments of the proposed chain (relationship → school belonging, and school belonging → prosocial behavior → academic achievement) were independently significant for both relational predictors. Supported and unsupported components of each hypothesis are summarized together in Table 3 to give equal visibility to both.

## 5. Discussion

The present study used structural equation modeling to examine the sequential mediating roles of school belonging and prosocial behavior in the effects of parent–adolescent and teacher–student relationships on adolescents’ academic achievement. Overall, the findings indicate that relational contexts are strongly associated with adolescents’ socio-emotional development, but their association with academic achievement was more limited and inconsistent across pathways than a uniformly indirect account would suggest. Both parent–adolescent and teacher–student relationships significantly predicted school belonging and prosocial behavior, and the proximal segment of the proposed sequential pattern was supported: the indirect effects of these relationships on prosocial behavior through school belonging were significant, and the link from school belonging to academic achievement through prosocial behavior was likewise significant. When the specific three-path serial indirect effect proposed for each relational predictor—relationship → school belonging → prosocial behavior → academic achievement—was tested directly, it reached statistical significance for teacher–student relationships (β = 0.027, 95% CI [0.004, 0.083]), although the effect was small in magnitude and the confidence interval lay close to zero at its lower bound, whereas the corresponding effect for parent–adolescent relationships did not reach significance (β = 0.016, 95% CI [0.000, 0.007]). The full sequential mediation hypothesis (H5) was therefore partially supported: the proposed mechanism received statistical support for the teacher–student pathway, but not for the parent–adolescent pathway, even though the constituent proximal links were independently significant for both. This pattern suggests that the proximal socio-emotional mechanism functions largely as hypothesized, and that the more distal indirect pathway from relational contexts to academic achievement, while statistically confirmed only for the teacher–student relationship, was weak and imprecisely estimated even where it was significant. This apparent tension—significant proximal mediation alongside a modest and inconsistently significant distal pathway—is not contradictory but reflects the additive nature of sequential mediation: each individual link in the proposed chain (relationship → belonging, belonging → prosocial behavior, prosocial behavior → achievement) was itself significant, yet combining three estimated paths into a single serial product compounds uncertainty, which is consistent with the small effect sizes and wide confidence intervals observed for both specific serial pathways. Accordingly, the present findings should be read as evidence for a plausible, theoretically coherent process operating at the proximal level, and for a tentative, source-specific extension of this process to academic achievement via the teacher–student pathway, rather than as confirmation that relational contexts uniformly exert a statistically reliable distal influence on academic achievement through this specific sequential mechanism. These findings are discussed below in relation to the existing literature.

Teacher–student relationships showed strong and significant effects on both school belonging and prosocial behavior. One plausible explanation is that teachers are among the closest social reference points for adolescents within the school context: as school becomes central to daily life during adolescence, teachers function not only as academic figures but also as sources of emotional and social support. Trust-based relationships with teachers may therefore facilitate students’ sense of acceptance and value at school, strengthening their sense of belonging. This interpretation is consistent with prior work identifying teacher–student relationships as one of the strongest predictors of school belonging [33,34,38] and as a shaping influence on students’ social behavior [23,25]. By contrast, teacher–student relationships did not show a significant direct effect on academic achievement, nor did their total indirect effect on academic achievement reach significance (Table 2). However, when the specific three-path serial pathway through school belonging and prosocial behavior was evaluated directly, it was statistically significant (β = 0.027, 95% CI [0.004, 0.083]; Table 3), even though this effect was small in magnitude and its confidence interval lay close to zero at the lower bound. Taken together, these results suggest that, within the present model, teacher–student relationships were linked to academic achievement specifically through this sequential socio-emotional mechanism, rather than through a direct association or through the aggregate of all indirect routes combined. This dissociation between a nonsignificant total indirect effect and a significant specific serial effect is not contradictory: the total indirect effect combines multiple routes of differing sign and precision, and can therefore remain nonsignificant even when one constituent pathway, evaluated on its own, is significant. Given that teacher–student relationships showed among the strongest effects on both school belonging and prosocial behavior in this model, this pattern is broadly consistent with expectations, although the modest size and imprecision of the specific serial effect indicate that it should be interpreted as tentative evidence of a distal mechanism rather than as a robust or firmly established one; replication with larger samples and longitudinal designs would help clarify the reliability of this pathway. This does not rule out the possibility that teacher–student relationships are also linked to academic achievement through a more direct academic channel, such as instructional support or classroom-specific practices, which were not assessed in the present study.

Findings regarding parent–adolescent relationships indicate that these relationships have significant, positive effects on school belonging and prosocial behavior. This may reflect the role of parental warmth, acceptance, and supportive communication in adolescents’ socio-emotional development; feelings of trust and acceptance experienced within the family may generalize to a comparable sense of belonging in the school environment, consistent with attachment theory’s account of how early relational experiences generalize to later social contexts [3,16]. These results align with prior evidence linking parental support to both prosocial behavior [11,12,14,15] and academic achievement [4,8]. The additional finding that parent–adolescent relationships had a significant direct effect on academic achievement suggests that parental influence on adolescents’ academic orientation may extend beyond purely socio-emotional pathways, consistent with the broader literature indicating that parental relationships shape adolescent development through both direct and indirect routes. By contrast, neither the total indirect effect of parent–adolescent relationships on academic achievement (Table 2) nor the specific serial pathway through school belonging and prosocial behavior (β = 0.016, 95% CI [0.000, 0.007]; Table 3) reached statistical significance. Taken together, these results suggest that, within the present model, the association between parent–adolescent relationships and academic achievement is best characterized as operating through a direct pathway rather than through the socio-emotional mechanisms examined here.

School belonging significantly predicted prosocial behavior, but its direct effect on academic achievement was not significant, suggesting that school belonging functions primarily as a mechanism supporting socio-emotional processes rather than directly enhancing academic performance. A student’s sense of acceptance and value at school may facilitate greater openness to social interaction and prosocial engagement, consistent with prior findings linking school belonging to prosocial behavior [23,47] and indicating that belonging’s influence on academic achievement is largely indirect [19,20,21]. The non-significant direct path from belonging to achievement observed here is consistent with the multidimensional nature of academic achievement, which is shaped by the interaction of numerous cognitive, motivational, and contextual variables beyond relational belonging. Notably, although the direct effect of school belonging on academic achievement was not significant, its indirect effect through prosocial behavior was significant, indicating that belonging is linked to achievement not directly but through prosocial behavior.

Finally, prosocial behavior had a significant, positive effect on academic achievement. Socially well-adjusted and cooperative students may participate more actively in learning processes; prosocial behaviors can enhance classroom interaction and support healthier relationships with teachers and peers, making the learning environment more supportive overall. This is consistent with evidence linking prosocial behavior to academic achievement [47,53,54] and with meta-analytic findings that social–emotional skills enhance academic adjustment [55]. This association may also partly reflect how academic achievement itself is assessed: because report card grades incorporate not only cognitive performance but also elements of classroom conduct, participation, and behavioral adjustment [62,63], teachers’ grading practices may implicitly reward the same cooperative and compliant behaviors captured by the prosocial behavior measure, which could inflate the observed association beyond a purely motivational or cognitive mechanism. At the same time, the comparatively modest size of this effect in the present study suggests that academic achievement cannot be explained by social behavior alone, and that cognitive ability, motivation, and environmental factors likely play substantial additional roles. The effect of prosocial behavior on academic achievement therefore appears meaningful but limited, and should be interpreted within a broader contextual framework rather than as a standalone predictor.

### 5.1. Theoretical and Practical Implications

Theoretically, the present study extends relational models of adolescent development by clarifying how interpersonal contexts relate to academic achievement. The findings suggest a differentiated pattern rather than a uniform one: parent–adolescent relationships were associated with academic achievement primarily through a direct pathway, whereas teacher–student relationships showed no significant direct association with academic achievement, but were linked to it through the specific sequential pathway involving school belonging and prosocial behavior, albeit weakly and imprecisely, despite their strong effects on school belonging and prosocial behavior themselves. School belonging, in turn, was associated with academic achievement not directly, but specifically through prosocial behavior. This refines the assumption that a sense of belonging translates directly into academic gains, and instead positions prosocial behavior as a key proximal mechanism linking relational contexts to achievement, consistent with recent findings that prosocial behavior can operate as a mediating mechanism linking contextual stressors to well-being and adjustment outcomes [44]. More broadly, these findings suggest that relational contexts are more reliably linked to adolescents’ socio-emotional development—school belonging and prosocial behavior—than to academic achievement itself, and that the specific route by which each relational context relates to achievement, differs by source: primarily direct for parent–adolescent relationships, and primarily through the sequential socio-emotional mechanism, albeit tentative, for teacher–student relationships. These patterns were observed within a Turkish adolescent sample, and their generalizability to other cultural contexts should be considered with caution. The relatively collectivistic orientation and the centrality of family and teacher authority within Turkish educational culture may shape the strength of the relational pathways examined here differently than in more individualistic educational systems; cross-cultural replication would help clarify whether the present sequential pattern reflects a broadly generalizable process or one that is, at least in part, culturally specific.

Several recommendations follow from these findings at both the research and practice levels. Future studies could use longitudinal designs to examine how the effects of parent–adolescent and teacher–student relationships on school belonging and prosocial behavior unfold over time, and in particular to re-examine the specific sequential pathway tested here (relationship → school belonging → prosocial behavior → academic achievement) with larger samples and repeated measures, given that the present cross-sectional test of this full pathway reached significance only for the teacher–student relationship, and even then yielded a small and imprecisely estimated effect, while the corresponding pathway for the parent–adolescent relationship did not reach significance. Considering academic achievement not only through traditional indicators such as school grades but also through process-oriented variables such as academic engagement, motivation, and self-regulation may further enhance the explanatory power of this model. From a practical standpoint, given the substantial influence of teacher–student relationships on school belonging and prosocial behavior observed here, social–emotional learning interventions that support teachers’ classroom interactions may be a productive focus for schools. Similarly, parent-awareness programs that strengthen supportive parenting practices could foster adolescents’ sense of belonging and prosocial behavior. Within schools, inclusive climate practices that help students feel accepted and valued are also indicated. Finally, given the observed role of prosocial behavior in academic achievement, programs that build cooperation, empathy, and helping behavior may support both students’ social adjustment and their academic development. These recommendations should nonetheless be regarded as plausible directions warranting further evaluation rather than as firmly established interventions, given that the present findings are correlational and cross-sectional; the modest direct effects, together with a distal sequential pathway that reached significance only for one of the two relational predictors and even then remained small in magnitude, suggest that such interventions alone are unlikely to substantially alter academic outcomes and should be considered as one component within a broader set of educational supports.

### 5.2. Limitations and Future Directions

Several limitations should be considered when interpreting these findings and in designing future research. First, because the study used a cross-sectional design, the relationships among variables cannot be interpreted as causal. Although structural equation modeling allows directional relationships to be specified and tested, the findings remain correlational, and the study cannot clarify how the relatively weak association between school belonging and academic achievement unfolds over time. Second, while assessing academic achievement through both subjective (perceived success) and objective (school grades) indicators is a strength of the design, report card grades are based on teacher assessment and incorporate behavioral elements beyond cognitive performance, which complicates the interpretation of results involving this indicator; this may partly explain the non-significant direct effect of belonging on academic achievement. Third, because all data were collected via self-report, common-method bias cannot be ruled out; in particular, prosocial behavior—a socially desirable construct—may have been overreported. Harman’s single-factor test suggested that a single dominant factor was unlikely to account for the shared variance among study measures, providing some reassurance on this point; nonetheless, this statistical check cannot fully substitute for a multi-informant design, and the possibility of shared-method variance remains a limitation. Fourth, the internal consistency of the study measures varied in adequacy. The parent–adolescent relationship scale (α = 0.82) and the school belonging scale (α = 0.77) demonstrated satisfactory reliability. The teacher–student relationship scale initially showed weaker psychometric performance in its full form; item-level inspection identified two items—Item 4 (“I am wary of the teachers at my school”) and Item 8 (“The teachers at my school are rude to me”)—that showed comparatively low factor loadings and reduced overall scale reliability. Removing these two items improved both internal consistency and model fit, yielding a final six-item form with adequate reliability (α = 0.84). This modification should be interpreted with appropriate caution: because the decision to remove these items was based on their performance within the same sample used to evaluate the resulting six-item measure, this reliability estimate reflects a data-dependent refinement rather than an independently validated psychometric structure. Notably, both excluded items were negatively worded (describing an unfavorable teacher relationship), which raises the possibility that their weaker performance partly reflected method effects associated with negatively phrased items rather than purely substantive content; this interpretation is offered tentatively and should not be treated as an alternative justification for their removal. The six-item form should therefore be treated as provisional pending replication or cross-validation in an independent sample before it is adopted as a standard measure in future research. By contrast, the prosocial behavior scale showed a comparatively low alpha (α = 0.64) that could not be attributed to a specific problematic item, suggesting a genuine limitation of this measure rather than an artifact correctable through item removal; findings involving prosocial behavior should therefore be interpreted with appropriate caution. Relatedly, confirmatory factor analyses indicated that the fit of the parent–adolescent relationship and school belonging measurement models was only marginally acceptable by conventional standards, which may have introduced additional imprecision into the structural estimates involving these constructs. Future research would benefit from psychometric refinement of the prosocial behavior measure, for example through the addition or revision of items, as well as from the use of alternative or supplementary indicators with stronger measurement properties, and from cross-validating the revised six-item teacher–student relationship scale in an independent sample.

Finally, the sample was limited to secondary school students from a single city, which restricts the generalizability of the findings; given the developmental characteristics of adolescence and the cultural context of schooling, findings may not generalize to other socio-cultural settings. In addition, the present model did not include several variables known to influence adolescent academic achievement, such as socioeconomic status, cognitive ability, broader school characteristics, and peer relationships; the omission of these potential confounders means that the associations reported here may partly reflect the influence of unmeasured variables, and future studies incorporating such controls would help clarify the unique contribution of the relational and socio-emotional mechanisms examined in the present model. Future research should address these limitations through longitudinal and multi-informant designs, the inclusion of process-oriented achievement indicators (e.g., academic engagement, motivation, self-regulation), the use of measures with stronger psychometric properties, and replication of the model across diverse socio-cultural and developmental contexts.

## 6. Conclusions

The present study examined how parent–adolescent and teacher–student relationships relate to adolescents’ academic achievement through school belonging and prosocial behavior. The findings indicate that relational contexts are strongly linked to adolescents’ socio-emotional development, but their association with academic achievement followed a differentiated rather than a uniformly indirect pattern: the parent–adolescent relationship was linked to academic achievement primarily through a direct pathway, and its hypothesized distal indirect pathway to achievement via school belonging and prosocial behavior was not statistically supported. The teacher–student relationship, by contrast, showed no significant direct association with academic achievement, but the specific sequential pathway through school belonging and prosocial behavior was statistically supported, although the effect was small in magnitude and imprecisely estimated. Testing the full sequential pathway directly (relationship → school belonging → prosocial behavior → academic achievement) therefore yielded statistical support for the teacher–student relationship but not for the parent–adolescent relationship. Notably, school belonging was associated with academic achievement not directly but through prosocial behavior, underscoring prosocial behavior as a key proximal mechanism in this process. Overall, within the correlational and cross-sectional scope of the present design, and given that several of the more distal associations examined here were either nonsignificant or, where significant, modest in magnitude, the results highlight the potential value of strengthening supportive parent and teacher relationships and of fostering school belonging and prosocial behavior as plausible directions for promoting adolescents’ positive development in school settings, while acknowledging that their specific contribution to academic achievement, as distinct from broader socio-emotional adjustment, remains to be more firmly established.

## Figures and Tables

**Table 1:** Descriptive Statistics and Correlation Coefficients for the Study Variables.

Variable	α	*M*	*SD*	Skew.	Kurt.	1	2	3	4	5
1. Parent–adolescent relationship	0.82	55.02	11.36	−0.07	−0.13	—				
2. Teacher–student relationship	0.84	16.92	3.80	−0.67	0.77	0.16 **	—			
3. School belonging	0.77	30.04	4.94	−0.23	−0.44	0.13 **	0.38 **	—		
4. Prosocial behavior	0.64	13.74	2.03	−1.13	1.50	0.14 **	0.38 **	0.29 **	—	
5. Academic achievement	—	86.81	9.24	−1.40	2.07	0.19 **	0.19 **	0.08	0.17 **	
6. Academic achievement	—	3.48	0.95	0.13	0.99	0.10 *	0.16 **	0.13 **	0.18 **	0.31 **

Note. N = 523. α = Cronbach’s alpha; correlations are Pearson coefficients. The internal consistency coefficients for the prosocial behavior scales (α = 0.64) fell below the conventional 0.70 threshold; associated findings should be interpreted with appropriate caution (see Section 5.2). * *p* < 0.05; ** *p* < 0.01.

**Table 2:** Standardized Indirect Effects with Bias-Corrected Bootstrap 95% Confidence Intervals.

Path	Effect	SE	LLCI	ULCI
Teacher–student relationship → School belonging → Prosocial behavior	0.099	0.046	0.025	0.213
Parent–adolescent relationship → School belonging → Prosocial behavior	0.055	0.029	0.012	0.136
Teacher–student relationship → Academic achievement (total indirect)	0.109	0.077	−0.036	0.269
Parent–adolescent relationship → Academic achievement (total indirect)	0.051	0.040	−0.025	0.137
School belonging → Prosocial behavior → Academic achievement	0.049	0.038	0.001	0.168

Note. Effect = standardized indirect effect; SE = standard error; LLCI and ULCI = lower and upper limits of the bias-corrected bootstrap 95% confidence interval (5000 resamples). Indirect effects whose confidence interval does not include zero are statistically significant.

**Table 3:** Summary of Hypothesis Testing Results.

Hypothesis	Paths Tested	β	*p*	Support Status
H1	Parent–adolescent relationship → School belonging	0.245	<0.001	Supported
	Teacher–student relationship → School belonging	0.444	<0.001	
H2	School belonging → Prosocial behavior	0.223	0.013	Supported
	Parent–adolescent relationship → Prosocial behavior (direct)	0.158	0.011	
	Teacher–student relationship → Prosocial behavior (direct)	0.358	<0.001	
H3	Prosocial behavior → Academic achievement	0.220	0.041	Partially supported
	Parent–adolescent relationship → Academic achievement (direct)	0.257	0.002	
	Teacher–student relationship → Academic achievement (direct)	0.129	0.169	
	School belonging → Academic achievement (direct)	0.019	0.857	
H4	Parent–adolescent relationship → School belonging → Prosocial behavior	0.055	0.010	Supported
	Teacher–student relationship → School belonging → Prosocial behavior	0.099	0.011	
H5	Parent–adolescent relationship → School belonging → Prosocial behavior → Academic achievement	0.016	-	Not supported
	Teacher–student relationship → School belonging → Prosocial behavior → Academic achievement	0.027	-	Supported

Note. β = standardized path coefficient (direct effects) or standardized indirect effect (H4 and H5); *p* values for H4 are based on bias-corrected bootstrap confidence intervals (5000 resamples), where a 95% CI excluding zero was treated as equivalent to *p* < 0.05. The same bias-corrected bootstrap criterion was applied to the specific three-path serial indirect effects tested for Hypothesis 5; these effects were estimated as defined parameters and are reported in the text (Section 4.3) rather than in Table 2, and support status for H5 was determined by this confidence interval. A hypothesis was classified as partially supported when some, but not all, of its constituent paths reached statistical significance. For H5, support status was determined using the bias-corrected bootstrap 95% confidence intervals for the specific three-path serial indirect effects reported in Table 2; Wald *p* values are therefore not reported.

## Data Availability

The datasets generated and/or analyzed during the current study are available from the corresponding author upon reasonable request. The data are not publicly available due to privacy and ethical restrictions related to the protection of participants’ personal information.
